# Exploring quantitative traits-associated copy number deletions through reanalysis of UK10K consortium whole genome sequencing cohorts

**DOI:** 10.1186/s12864-023-09903-3

**Published:** 2023-12-18

**Authors:** Sejoon Lee, Jinho Kim, Jung Hun Ohn

**Affiliations:** 1 Precision Medicine Center, Future Innovation Research Division, Seoul National University Bundang Hospital, 173-82, Gumi-ro, Bundang-gu, Seongnam, Gyeonggi-do 13620 South Korea; 2 Department of Pathology, Seoul National University Bundang Hospital, 173-82, Gumi-ro, Bundang-gu, Seongnam, Gyeonggi-do 13620 South Korea; 3 Department of Laboratory Medicine, Seoul National University Bundang Hospital, 173-82, Gumi-ro, Bundang-gu, Seongnam, Gyeonggi-do 13620 South Korea; 4 Department of Internal Medicine, Seoul National University Bundang Hospital, 173-82, Gumi-ro, Bundang-gu, Seongnam, Gyeonggi-do 13620 South Korea; 5 Department of Internal Medicine, College of Medicine, Seoul National University, 103, Daehak-ro, Jongno-gu, Seoul, 03080 South Korea

**Keywords:** Structural variation, UK10K, Genome-wide association study

## Abstract

**Objectives:**

We performed comprehensive association analyses of common high-confidence gnomAD-reported copy number deletions (CNDs) with 60 quantitative traits from UK10K consortium WGS data.

**Methods:**

The study made use of data generated by the UK10K Consortium. UK10K consortium WGS data consist of TwinsUK (n = 1754, middle-aged females) and ALSPAC (n = 1867, birth to adolescence) cohorts. UK10K consortium called 18,739 CNDs (hg19) with GenomeSTRiP software. After filtering out variants with minor allele frequency < 0.05 or HWE P < 1.0 × 10^− 6^, 1222 (TwinsUK) and 1211 (ALSPAC) CNDs remained for association analyses with 60 normalized quantitative traits.

**Results:**

We identified 23 genome-wide significant associations at 13 loci, among which 2 associations reached experiment-wide significance. We found that two common deletions in chromosome 4, located between *WDR1* and *ZNF518B* (23.3 kb, dbVar ID:nssv15888957, 4:10211262–10,234,569 and 9.8 kb, dbVar ID:nssv15888975, 4:10392422–10,402,191), were associated with uric acid levels (P = 5.23 × 10^− 11^ and 2.29 × 10^− 8^, respectively). We also discovered a novel deletion spanning chromosome 18 (823 bp, dbVar ID: nssv15841628, 8:74347187–74,348,010) associated with low HDL cholesterol levels (P = 4.15 × 10^− 7^). Additionally, we observed two red blood cell traits-associated loci with genome-wide significance, a 13.2 kb deletion in 7q22.1 (nssv15922542) and a 3.7 kb deletion in 12q24.12 (nssv15813226), both of which were located in regions previously reported to be associated with red blood cell traits. Two deletions in 11q11 (nssv15803200 and nssv15802240), where clusters of multiple olfactory receptor genes exist, and a deletion (nssv15929560) upstream to *DOCK5* were associated with childhood obesity. Finally, when defining Trait-Associated copy number Deletions (TADs) as CNDs with phenotype associations at sub-threshold significance (P < 10^− 3^), we identified 157 (97.5%) out of 161 TADs in non-coding regions, with a mean size of 4 kb (range: 209 − 47,942 bp).

**Conclusion:**

We conducted a reanalysis of the UK10K Whole Genome Sequencing cohort, which led to the identification of multiple high confidence copy number deletions associated with quantitative traits. These deletions have standard dbVar IDs and replicate previous findings, as well as reveal novel loci that require further replication studies.

**Supplementary Information:**

The online version contains supplementary material available at 10.1186/s12864-023-09903-3.

## Introduction

Copy number variations (CNVs) are unbalanced rearrangements of DNA segments that involve at least 50 nucleotides with gain (duplication) or loss (deletion) of DNA contents [1, 2]. It is estimated that 4.8–9.5% of the genome contributes to CNVs and encompass more polymorphic base pairs than single nucleotide variations (SNPs) [3]. CNVs are reported to have a global effect on the transcriptome [4, 5] and contribute to various diseases and traits through numerous mechanisms disrupting protein-coding genes and *cis*-regulatory regions [6].

Microarrays including comparative genomic hybridization (CGH) and SNP-based arrays have been the primary approaches for CNV detection. However, with the advent of next-generation sequencing (NGS) technologies, new tools can detect CNVs from NGS data [7]. Compared to microarrays, NGS can detect smaller variants and have both higher sensitivity and the ability to provide accurate sequence-level breakpoint resolution, whereas array-based approaches have a limited resolution capacity [8].

Recently, a population reference for medical or population genetic studies, the Genome Aggregation Database (gnomAD), started to provide the reference of sequence-resolved structural variations like CNVs constructed from 14,891 whole genomes across diverse global populations [9]. Although whole genome sequencing (WGS) is rapidly becoming the predominant technology in large-scale human disease studies or biobank-based population cohort studies [10], few studies exist that investigated the association of high-confidence SVs from WGS with diseases or traits [11].

The UK10K consortium is one of the earliest large-scale WGS-based cohorts [12]. WGS was performed on a total of 3781 participants in two UK cohorts, the TwinsUK cohort and the ALSPAC cohort. TwinsUK is the UK’s largest twin cohort, comprising more than 14,000 twins who were predominantly female (82%) and middle-aged with the average of 59 years [13] and the ALSPAC cohort is a birth cohort in which more than 14,000 pregnant women and their children were followed for about 20 years from birth and health information from childhood to adolescence was collected [14].

In the present study, we performed a comprehensive association study of common high confidence copy number deletions (CNDs) reported in gnomAD with 60 distinct quantitative traits based on the UK10K consortium WGS cohort data. The traits were grouped into several broad classes such as lipid and glucose metabolism, body composition, cardiovascular health, serum chemistry, lung function, etc. We were able to replicate previously reported associations of CNDs with quantitative traits and identified new CNDs that showed associations with traits. Our findings suggest that incorporating structural variations in association studies could broaden our understanding of the connections between genotypes and phenotypes.

## Methods

### Whole genome sequencing and phenotype data of UK10K project

The access to whole genome sequencing and phenotype data of UK10K project (https://www.uk10k.org) was granted on 14th April 2018 (request ID:6410). Data were downloaded for 1754 subjects in TwinsUK cohort and 1867 subjects in ALSPAC (Avon Longitudinal Study of Parents and Children) cohort.

In the TwinsUK and ALSPAC cohorts, 48 and 42 unique phenotypes were identified, with 30 overlapping phenotypes between them (see Supplementary table S1). These phenotypes or traits were linked to various biological functions, including lipid and glucose metabolism, body composition, pulmonary function, cardiovascular health, blood cell composition, and serum biochemistry.

The TwinsUK cohort primarily consisted of adult female twins with phenotypes measured at 56.0 (standard deviation = 12.3) years of age on average. On the other hand, in ALSPAC cohort, phenotypes were longitudinally collected from childhood to adolescence. Specifically, glucose metabolic traits such as fasting glucose, insulin levels, insulin secretory function (HOMA-Beta), and insulin resistance (HOMA-IR) [15] were measured at 15 years of age. Lipid traits like total cholesterol, VLDL, HDL, LDL, triglycerides, APO-A1, and APO-B were measured at 9 years of age. Leptin, adiponectin, CRP, homocysteine, and hemoglobin were profiled at age 9. Pulmonary function tests were carried out at 8 years of age and blood pressures were measured at 9 years of age and heart rate was measured at 13 years of age. Body mass index (BMI) was serially measured at age 7, 9, 13, and 15 years of age and other body compositions like total fat mass and total lean mass by dual-energy x-ray absorptiometry (DEXA), and waist-to-hip ratio were measured at age 9.

### Copy number deletion dataset and annotation

Using whole genome sequencing data at a mean depth ×7, deletions ranging in size from 100 bp to 1 Mb were identified by UK10K consortium using the GenomeSTRiP(version1.04.1068) software [16] as described in the UK10K consortium flagship paper [12]. A total of 18,739 copy number deletions profiled for each cohort were downloaded in multi-sample vcf files (Supplementary table S2). Chromosome positions followed hg19 version of human reference genome. We annotated copy number deletions with VEP [17] and AnnotSV softwares [18, 19].

### Association analysis

We excluded copy number deletions on sex chromosomes and retained 18,056 variants. Further, we filtered out variants with a minor allele frequency < 0.05 or Hardy-Weinberg Equilibrium P < 1.0 × 10 ^− 6^ to focus our analysis on common variants. This resulted in a total of 1222 and 1211 common copy number deletions for association analysis in the TwinsUK and ALSPAC cohorts, respectively (Supplementary table S3). We applied the same filtering criteria for the association study of single nucleotide variations (SNV) and small insertions/deletions (Indel) with more than 5 million SNVs and Indels in each cohort. Prior to conducting association analysis, continuous phenotype variables were transformed by rank-based inverse normal transformation. We performed GWAS across all normalized traits adjusting for age and sex and assumed additive, dominant, or recessive genetic models (see Supplementary table S4 for GWAS summary statistics for CNDs). Hail version 0.2 software (https://github.com/hail-is/hail) was used for variant filtering and association analyses. After association analysis, we restricted analysis to CNDs with confirmed and comparable population allele frequencies reported in gnomAD SV database [9] and reported in dbVar [20]. In the meta-analysis of the two cohorts for 30 common phenotypes, METAL was used to conduct a fixed-effects meta-analysis and p values for association were combined [21]. Local association plot was generated with LocusZoom [22]. Other statistical analyses were conducted with R v4.0.2 (https://www.r-project.org/). Network diagrams were drawn with Cytoscape software [23].

## Results

### Association analysis of common copy number deletions

The genome-wide significance threshold was set as 4.09 × 10^− 5^ and 4.13 × 10^− 5^ after Bonferroni correction at level α = 0.05 accounting for 1222 and 1211 variables in TwinsUK and ALSPAC cohorts, respectively. Furthermore, the experiment-wide significance threshold was set as 2.84 × 10^− 7^ and 3.28 × 10^− 7^ correcting for 48 and 42 independent variables within TwinsUK and ALSPAC cohorts, respectively, and three assumed models of genetic inheritance.

**Fig. 1:**
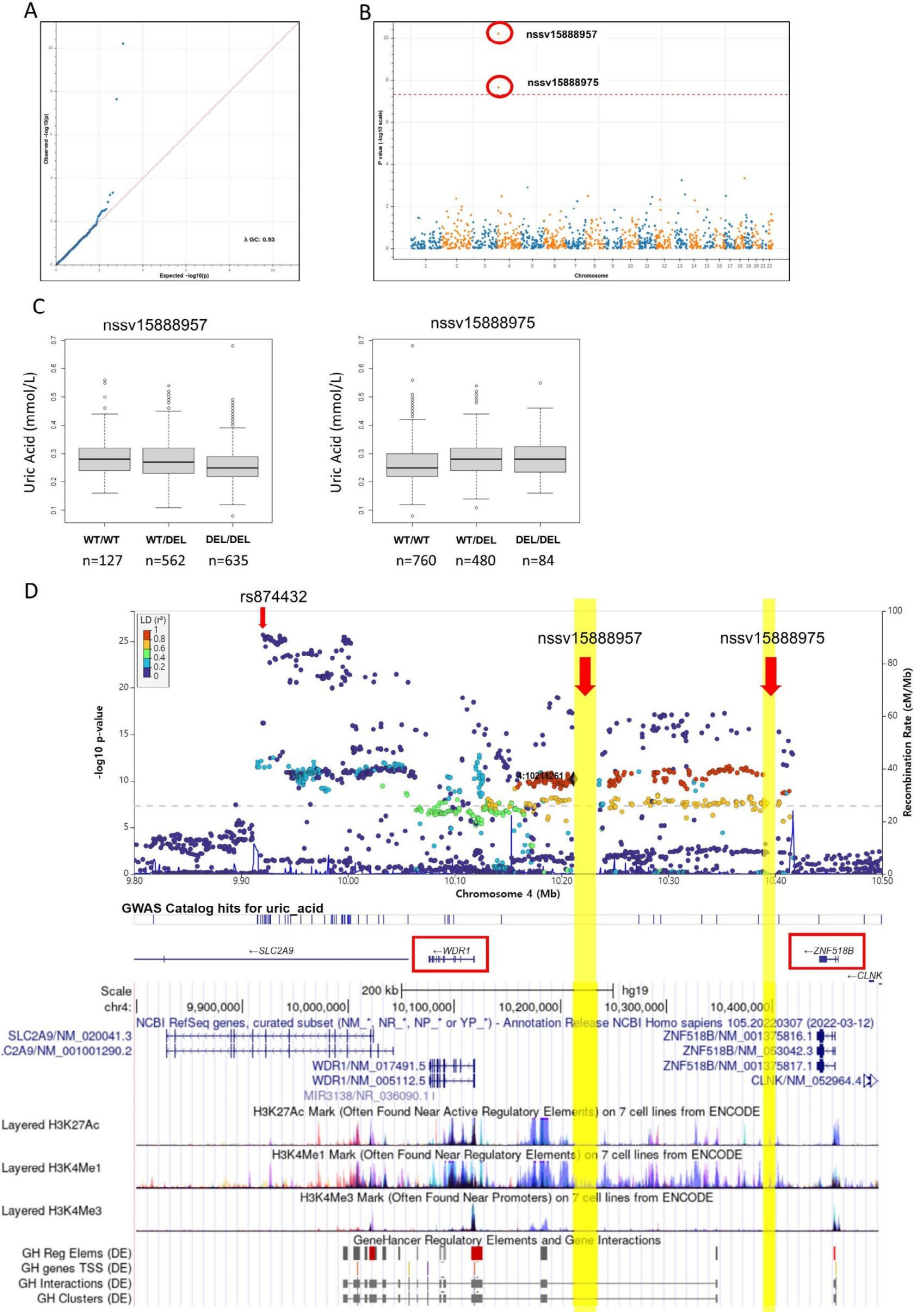
(**A**) QQ plot and (**B**) Manhattan plot of uric acid associated copy number deletions. Two deletions passing experiment-wide significance are marked with red circles. (**C**) nssv15888957 and nssv15888975 copy number deletions are associated with lower and higher serum uric acid levels, respectively. (**D**) Fine mapping analysis of deletions and SNVs in linkage disequilibrium with nssv15888957. Variants were colored by the category of linkage disequilibrium R^2^ values with respect to nssv15888957. It demonstrates that nssv15888957 forms a 254 kb-ranging haplotype block in strong linkage disequilibrium with 224 SNVs. Upper panel shows LocusZoom plot and lower panel is from UCSC genome browser annotated with histone marks and GeneHancer reported regulatory elements

In the TwinsUK cohort, two loci associated with uric acid levels surpassed the experiment-wide significance threshold (Table 1). A common 23.3 kb deletion in chromosome 4 (dbVar ID:nssv15888957, 4:10211262–10,234,569) with deletion allele frequency 0.69 (0.68 among gnomAD Europeans) was associated with reduced uric acid levels, whereas adjacent common 9.8 kb deletion in chromosome 4 (dbVar ID:nssv15888975, 4:10392422–10,402,191) with deletion allele frequency 0.24 (0.25 among gnomAD Europeans) was associated with elevated uric acid levels (Fig. 1). A GWAS including SNVs and Indels with uric acid levels in TwinsUK cohort confirmed the significant peak on the chromosome 4 (Supplementary Figure S1). These two copy number deletions exhibited moderate linkage disequilibrium (R^2^ = 0.69) and placed between *WDR1*(WD Repeat Domain 1) and *ZNF518B* (Zinc Finger Protein 518B) genes near the urate transporter *SLC2A9* (Solute Carrier Family 2 Member 9) gene which is reported to be associated with uric acid levels and gout. Among common SNVs in the region, the A allele of rs874432, an intron variant of *SLC2A9*, was associated with lower uric acid levels with the most statistical significance (P = 1.98 × 10^− 26^). After adjusting for age and rs874432, nssv15888957 (adjusted P = 3.69 × 10^− 4^) and nssv15888975 (adjusted P = 2.26 × 10^− 3^) remained associated with serum uric acid levels at nominal significance. At combined analysis with adjacent SNVs in the region, nssv15888957 formed a 254 kb-ranging haplotype block in strong linkage disequilibrium with 224 SNVs (R^2^ > 0.8)(Fig. 1). The haplotype block is placed in active regulatory element region that harbors two enhancers, GH04J010179 (4:10180825–10,188,676) and GH04J010344(4:10346574–10,348,589), curated in GeneHancer database, that target *SLC2A9*, *WDR1*, and *ZNF518B* in many cell types including kidney and urothelial cells [24].

**Table 1:** Copy number deletions associated with traits at experiment-wide or genome-wide significance

Locus chr:position (dbVar ID)	Trait(unit)	Subject No.	Beta(S.E)**	P***	Genetic model	Cohort	Cohort MAF (Homozygote count:Ref,Del)	gnomAD MAF (gnomAD ID) ****	RefSeq Gene	annotation
4:10211262–10,234,569 (nssv15888957)	Uric Acid (mmol/L)	1324	-0.02(0.00)	5.23E-11^#^	recessive	TwinsUK	0.69(166,842)	0.68	WDR1/ ZNF518B	Enhancer
4:10392422–10,402,191 (nssv15888975)	Uric Acid (mmol/L)	1324	0.02(0.00)	2.29E-08^#^	additive	TwinsUK	0.24(1008,105)	0.25	WDR1/ ZNF518B	Enhancer
18:74347187–74,348,010 (nssv15841628)	ApoA1(g/L)/	1470	-0.13(0.03)	1.87E-05	recessive	TwinsUK	0.29(865,126)	0.26	LINC01927	Intron 3
	HDL(mmol/L)	1718	-0.22(0.04)	4.15E-07						
2:166014696–166,016,748 (nssv15872979)	Leptins(ng/mL)	964	-3.24(1.03)	4.33E-06	dominant	TwinsUK	0.11(1379,18)	0.12	SCN3A	Intron 9
4:98354274–98,359,233 (nssv15893432)	Total lean mass (kg)	1721	-1.39(0.28)	1.42E-06	dominant	TwinsUK	0.14(1280,31)	0.13	STPG2-AS1	Intron 1
	Weight(kg)	1752	-3.34(0.72)	1.36E-05						
7:100327594–100,340,799 (nssv15922542)	MCH(pg/cell)	1575	-0.37(0.09)	1.68E-05	dominant	TwinsUK	0.29(866,125)	0.24	ZAN	LOF(txStart-intron7)
12:111976297–111,979,993 (nssv15813226)	MCV(fl.)	1575	1.19(0.28)	1.56E-05	dominant	TwinsUK	0.10(1407,17)	0.07	ATXN2	Intron 5
6:134589734–134,594,596 (nssv15913056)	Sodium (mmol/L)	1724	3.23(0.98)	1.74E-05	recessive	TwinsUK	0.10(1403,14)	0.11	SGK1	Intron 1
11:49759283–49,760,872 (nssv15803200)	Body mass index (kg/m^2^,15 year)	1608	0.93(0.20)	1.78E-05	dominant	ALSPAC	0.10(1506,18)	0.09	GRM5P1	Intron 2
	Total Fat Mass (kg,9 year)	1703	1.39(0.27)	3.58E-06	Additive					
	Truncal Fat Mass (kg,9 year)	1703	0.64(0.13)	4.56E-06	Additive					
11:55031591–55,038,554 (nssv15802240)	Body mass index (kg/m^2^,15 year)	1608	1.03(0.20)	1.72E-06	dominant	ALSPAC	0.11(1486,25)	0.11	TRIM48	LOF(intron1-exon6)
	Total Fat Mass (kg,9 year)	1703	1.29(0.26)	1.31E-05	additive					
	Truncal Fat Mass (kg,9 year)	1703	0.60(0.13)	1.66E-05	additive					
8:24972435–24,990,944 (nssv15929560)	Hip circumference (cm,9 year)	1811	1.49(0.32)	2.75E-06	dominant	ALSPAC	0.34(826,218)	0.35	DOCK5	Intergenic
	Total Fat Mass (kg,9 year)	1703	0.96(0.23)	2.63E-06						
	Truncal Fat Mass (kg,9 year)	1703	0.45(0.11)	2.15E-06						
	Weight (kg,9yrs)	1812	1.45(0.33)	1.48E-05						
4:172374416–172,379,428 (nssv15893806)	Triglycerides (mmol/L,9 year)	1497	0.16(0.06)	1.95E-05	dominant	ALSPAC	0.75(104,1034)	0.74		Intergenic
	VLDL (mmol/L,9 year)	1497	0.07(0.03)	1.86E-05						
1:234318630–234,319,762 (nssv15849193)	LDL (mmol/L,9 year)	1497	0.16(0.04)	3.47E-05	additive	ALSPAC	0.09(1543,11)	0.10	SLC35F3	Intron 2

*Reference sequence is GRCh37.

**calculated by GWAS of traits before variable transformation

***P values are adjusted for age and sex after rank-based normal transformation of traits

****gnomAD SV allele frequency in Europeans

#Experiment-wide significant

In addition to experiment-wide significant associations, there were 21 genome-wide significant associations for 11 loci in two cohorts in different genetic models (Table 1). At TwinsUK cohort, there were 8 genome-wide significance associations for 6 copy number deletions.

**Fig. 2:**
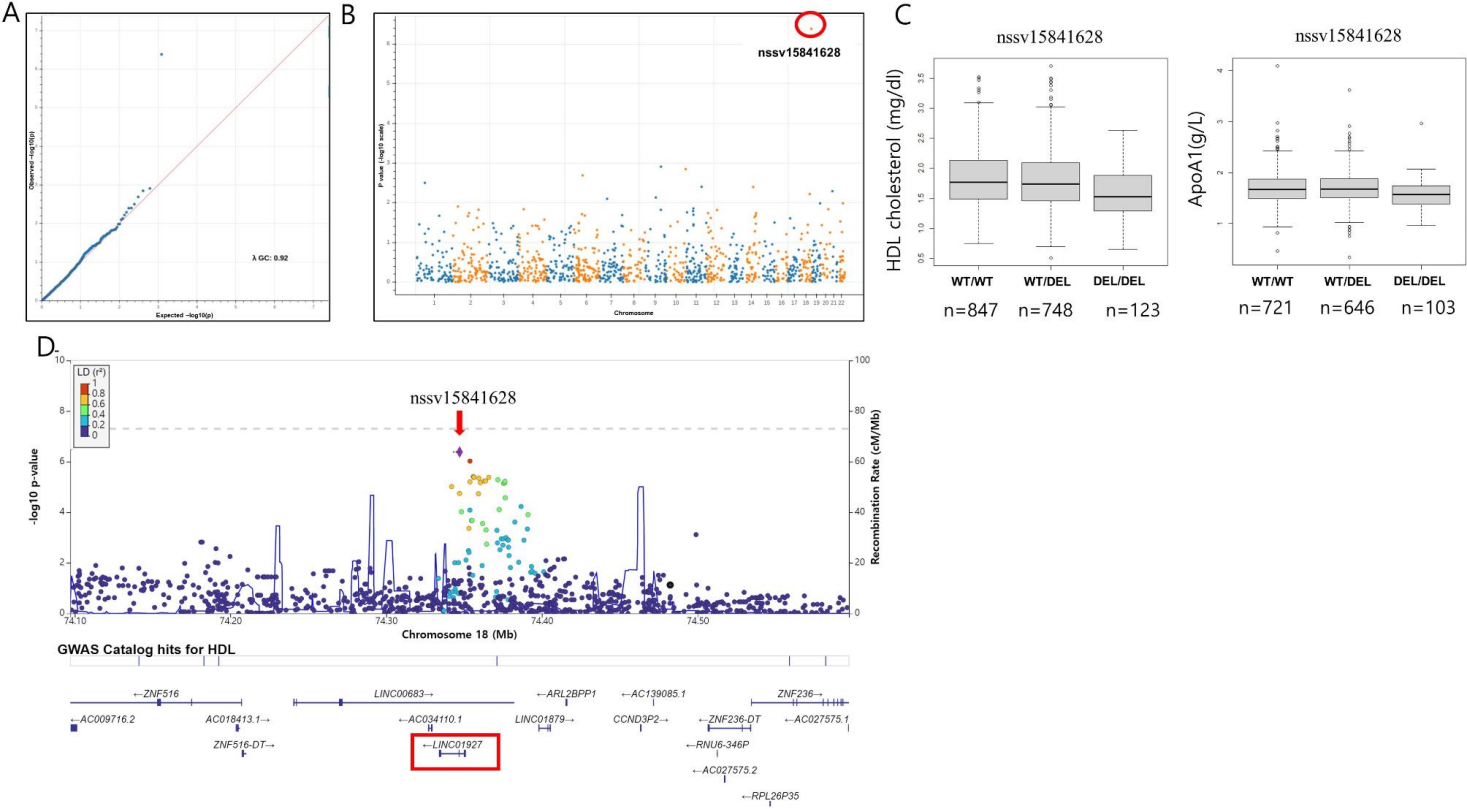
(**A**) QQ plot and (**B**) Manhattan plot of HDL cholesterol associated copy number deletions. nssv15841628 is marked with red circle. (**C**) nssv15841628 deletion is associated with lower HDL and ApoA1 levels. (**D**) Fine mapping analysis of deletions and SNVs in linkage disequilibrium with nssv15841628. Variants were colored by the category of linkage disequilibrium R^2^ values with respect to nssv15841628. The start position of nssv15841628 is marked with diamond

A common (deletion allele frequency 0.29) 823 bp deletion that spans on chromosome 18 (dbVar ID: nssv15841628, 18:74347187–74,348,010) was newly associated with low HDL cholesterol and ApoA1 levels at genome-wide significance, assuming recessive inheritance (Table 1). This deletion is located in intron 3 of lncRNA, LINC01927 (Fig. 2) and GWAS of SNVs and Indels with HDL levels also showed sub-threshold peak in the region (Supplementary Figure S2). Low HDL cholesterol < 40 mg/dL (1.034mmol/L) is a well-known major cardiovascular risk factor, and we categorized the subjects with HDL < 1.034mmol/L according to the number of deletion alleles. Subjects with two deletion alleles had higher proportion of subjects with HDL < 1.034mmol/L versus the rest with odd ratio = 1.90.

A 5 kb deletion in *STPG2* and *STPG2-AS1* (dbVar ID: nssv15893432, 4:98354274–98,359,233) was associated with lower total lean mass (Table 1 and supplementary figure  S3). nssv15893432 was not only associated with total lean mass but also lower BMI (P = 2.73 × 10^− 3^, β=-0.16), height (P = 1.04 × 10^− 3^, β=-0.17), FEV1(P = 4.09 × 10^− 3^, β=-0.12) and FVC(P = 4.24 × 10^− 3^, beta=-0.13) suggesting the link between poor lung function and sarcopenia [25].

Two red blood cell traits-associated copy number deletions were found near SNV loci with previously reported associations with red cell traits [26]. A 13.2 kb deletion in 7q22.1 with loss of function on *ZAN* (Zonadhesin) gene and downstream of *EPO* gene was associated with MCH. Another 3.7 kb deletion in 12q24.12 associated with MCV is found in intron 5 of *ATXN2* (Ataxin 2) gene which is associated with multiple red cell traits (Table 1 and supplementary figures S4-S5).

It is of note that a common deletion (dbVar ID: nssv15913056, 6:134589734–134,594,596) in intron 1 of *SGK1* (Serum/Glucocorticoid Regulated Kinase 1) which is involved in sodium regulation was associated with higher sodium levels (P = 1.74 × 10^− 5^, Supplementary figure S6).

**Fig. 3:**
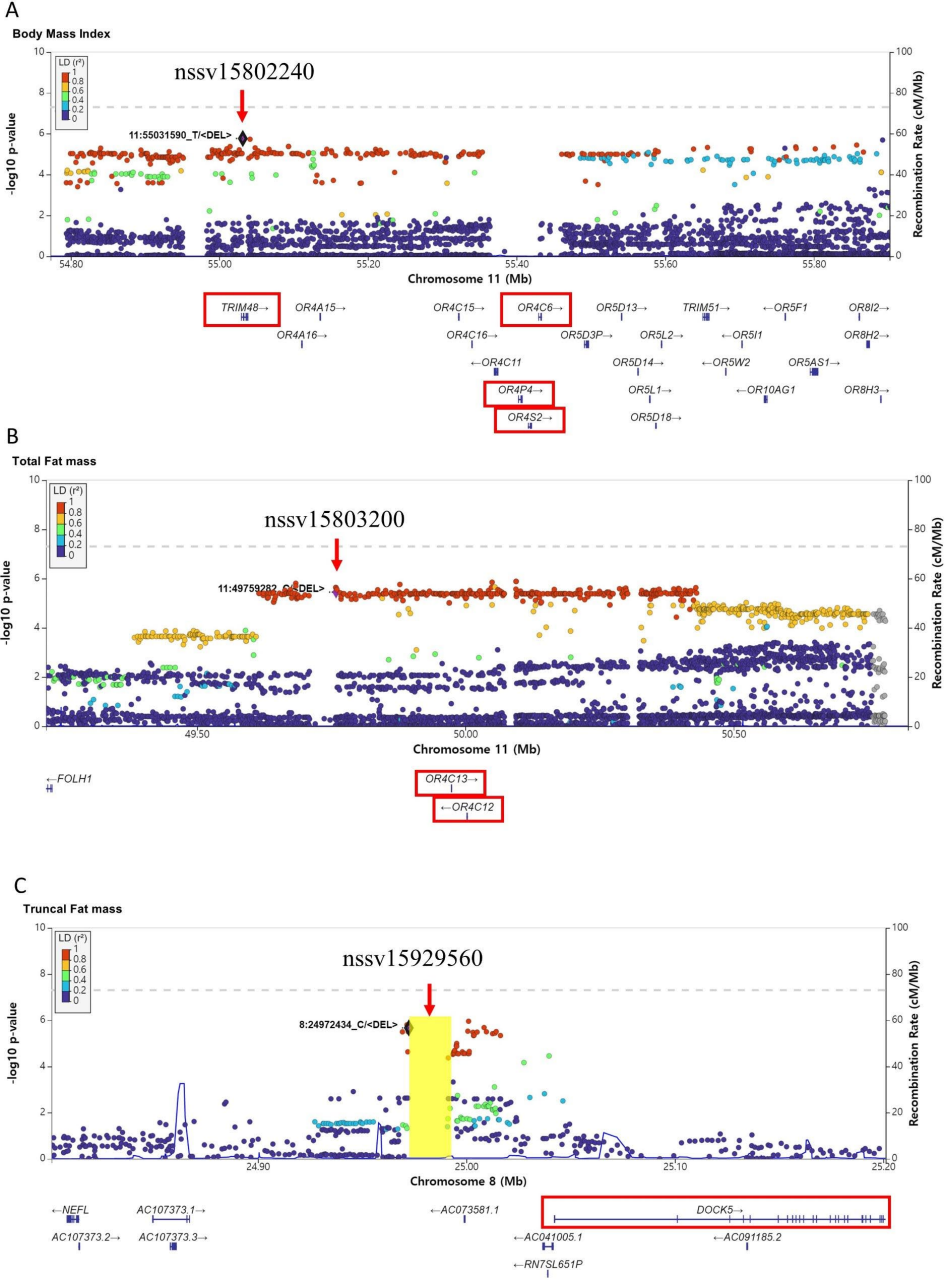
Fine mapping analysis of childhood obesity associated deletions. Variants were colored by the category of linkage disequilibrium R^2^ values with respect to copy number deletions. Haplotypes including SNVs in linkage disequilibrium with nssv15802240 associated with BMI (**A**) and nssv15803200 associated with total fat mass (**B**) formed a long haplotype block, encompassing multiple olfactory receptor genes in 11q11 region. (**C**) nssv15929560 associated with truncal fat mass is in upstream of *DOCK5* gene. The start positions of deletions are marked with diamonds

In the ALSPAC cohort, 13 genome-wide significance associations were found for 5 copy number deletions (Table 1). A 6.96 kb copy number deletion on chromosome 11 (dbVar ID: nssv15802240, 11:55031591–55,038,554) with loss of E3 ubiquitin-protein ligase *TRIM48* (tripartite motif containing 48) gene was associated with childhood obesity (Table 1). nssv15802240 was in strong linkage disequilibrium with adjacent SNVs and formed a long haplotype block harboring multiple genes in olfactory receptor gene cluster. The block contains a copy number deletion involving *OR4P4*, *OR4S2*, and *OR4C6* genes (Fig. 3) which is associated with early extreme obesity in previous studies using genotyping arrays and suggesting the link between olfactory dysfunction and obesity [27]. In the present study with whole genome sequencing, the region is a 79.6 kb common copy number deletion (dbVar ID:nssv15802291, 11:55364273–55,443,871, gnomAD deletion allele frequency 0.27 in Europeans) that spans olfactory receptor family 4 genes (*OR4C11*, *OR4P4*, *OR4S2*, and *OR4C6*) and was also nominally associated with early obesity (BMI at 15 years of age, β = 0.11, dominant, P = 3.54 × 10^− 2^) in the ALSPAC cohort. We also found a 1.59 kb copy number deletion (dbVar ID: nssv15803200, 11:49759283–49,760,872) within intron 2 of *GRM5P1*(GRM5 pseudogene 1) associated with early obesity. It also formed a long-ranging haplotype block with adjacent SNVs (Fig. 3) that involves olfactory receptor family 4 genes (*OR4C12* and *OR4C13*).

Another copy number deletion associated with early central obesity (truncal fat mass) was an 18.5 kb deletion in intergenic region on chromosome 8 (dbVar ID: nssv15929560) that was placed upstream of *DOCK5* (Dedicator Of Cytokinesis 5) gene (Fig. 3). *DOCK5* has been reported associated with severe obesity and regulate hepatic insulin resistance [28, 29].

A 1.1 kb deletion on chromosome 1 (dbVar ID: nssv15849193, 1:234318630–234,319,762) that disrupts the thiamine transporter *SLC35F3* (Solute Carrier Family 35 Member F3) was associated with higher LDL cholesterol in childhood, suggesting the link between thiamine deficiency and dyslipidemia (supplementary figure S7).

### Meta-analysis and sub-threshold associations replicated in the other cohort

Although phenotypes in children and adult women may have different characteristics, we performed meta-analysis for 30 quantitative traits shared by the two cohorts from GWAS summary statistics. Two loci were discovered surpassing the Bonferroni-corrected genome-wide significance threshold at combined meta-analysis p value (Table 2). A 3 kb deletion in *GUSBP1* (GUSB Pseudogene 1) enhancer on chromosome 5 (dbVar ID: nssv15898820, 5: 21611834–21614796) was associated with waist circumference and total fat mass and a 0.8 kb deletion on chromosome 3 (dbVar ID: nssv15883024, 3:141572125–141572896) in enhancer region targeting multiple genes was associated with anemia. Additionally, we also sought to find sub-threshold associations (P < 10^− 3^) that are replicated in the other cohort as traits can persist regardless of age and found 4 sub-threshold associations for 4 copy number deletions replicated in the other cohort (Table 2). The association of 4.7 kb deletion in 3’-UTR region of *FAM149A* gene (dbVar ID: nssv15897230, 4:187093507–187,098,168) with reduced lung function (FVC and FEV1) in adult women was replicated in childhood cohort. It is interesting that the deletion was associated with lower total lean mass (P = 3.47 × 10^− 3^) and body mass indices (BMI_7, P = 1.04 × 10^− 3^; BMI_9, P = 7.13 × 10^− 3^; BMI_13, P = 2.48 × 10^− 2^; BMI_15, P = 7.30 × 10^− 3^) in children, suggesting the genetic link between childhood sarcopenia and adult poor lung function (supplementary figure S8) [30].

**Table 2:** Copy number deletions with sub-threshold associations with traits replicated at the other cohort

Position*	Trait(unit)	Subject No.	Beta(S.E) _discovery_ **	P_discovery_/ P_replication_***	P_meta−analysis_	Genetic model	Cohort_discovery_	Cohort_discovery_ MAF (Homozygote count:Ref,Del)	gnomAD MAF (gnomAD ID) ****	RefSeq Gene	annotation
4:187093507–187,098,168 (nssv15897230)	FVC(L)	1,606	-0.06(0.02)	8.03E-04/ 4.92E-02	1.62E-04	additive	TwinsUK	0.56(332,546)	0.63	FAM149A	3’UTR(exon14-txEnd)
5:21611834–21,614,796 (nssv15898821)	Total Fat Mass (kg,9 year)	1,703	1.26(0.30)	1.79E-04 (2.52E-05^#^)/ 3.79E-02	3.88E-05^##^	additive	ALSPAC	0.08(1574,12)	0.08	GUSBP1	Enhancer
	Waist Circumference (kg)	1,812	1.72(0.44)	8.18E-04/ 4.35E-03	1.10E-05^##^						
3:141572125–141,572,896 (nssv15883024)	Hemoglobin (g/L,9 year)	1,524	-2.28(0.61)	1.82E-04/ 1.10E-02	9.11E-06^##^	additive	ALSPAC	0.09(1559/17)	0.08	ATP1B3;GK5 ;GRK7;RASA2;RNF7;TRPC1;XRN1;ZBTB38	Enhancer
4:172988628–172,992,939 (nssv15893853)	Heart rate (/min,13 year)	1,597	-1.63(0.42)	9.10E-05/ 4.43E-02	9.02E-05	additive	ALSPAC	0.49(476,454)	0.51	GALNTL6	Intron 2
1:243782752–243,783,761 (nssv15850019)	HOMA beta	1,580	17.16(9.07)	7.47E-04/ 4.96E-02	1.29E-04	dominant	TwinsUK	0.69(163,844)	0.66	AKT3	Intron 5
2:123364888–123,365,356 (nssv15869944)	TRIG (mmol/L,9 year)	1,497	-0.10(0.03)	2.69E-04/ 4.55E-02	7.83E-05	recessive	ALSPAC	0.63(242,712)	0.62		Intergenic

*Reference sequence is GRCh37.

**calculated by GWAS of traits before variable transformation

***P values are adjusted for age and sex after rank-based normal transformation of traits

****gnomAD SV allele frequency in Europeans

# untransformed, ## Bonferroni-corrected genome-wide significant

### Trait-associated copy number deletions (TAD)

**Fig. 4:**
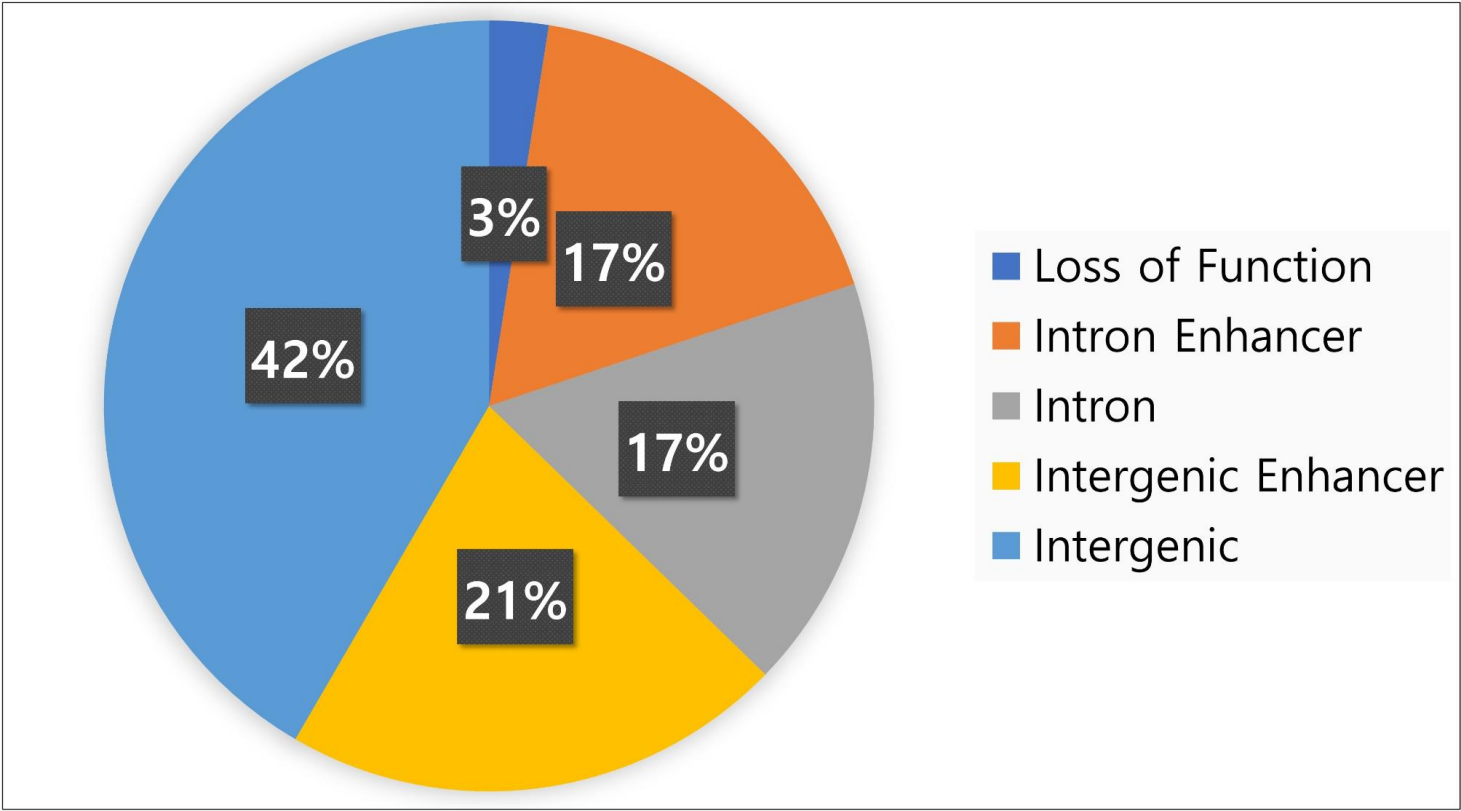
Genomic impact of 161 Trait-Associated copy number Deletions (TADs). Most (97.5%) TADs were found in non-coding regions. Out of 157 deletions in non-exon regions, 62 (39.5%) TADs were annotated to have enhancer activity

We defined Trait-Associated copy number Deletions (TADs) as copy number deletions associated with any phenotypes with at least sub-threshold significance (P < 10^− 3^). Out of 239 associations (Supplementary table S5), we discovered 161 Trait-Associated copy number Deletions (TADs) in the two cohorts (Supplementary table S6). The mean size of TADs was 4 kb (range: 209 − 47,942 bp). Overall, 157 (97.5%) out of 161 TADs were found in non-coding regions (Fig. 4). There were 60 TADs annotated in gene regions with 56 TADs in introns and only 4 TADs resulting in loss of function deletions of coding regions. Out of 56 TADs in intron regions, 28 TADs were annotated to have enhancer activity. Additionally, among 101 TADs in intergenic regions, 34 deletions were located in enhancer regions.

### Pleiotropy of trait-associated copy number deletions (TADs)

**Fig. 5:**
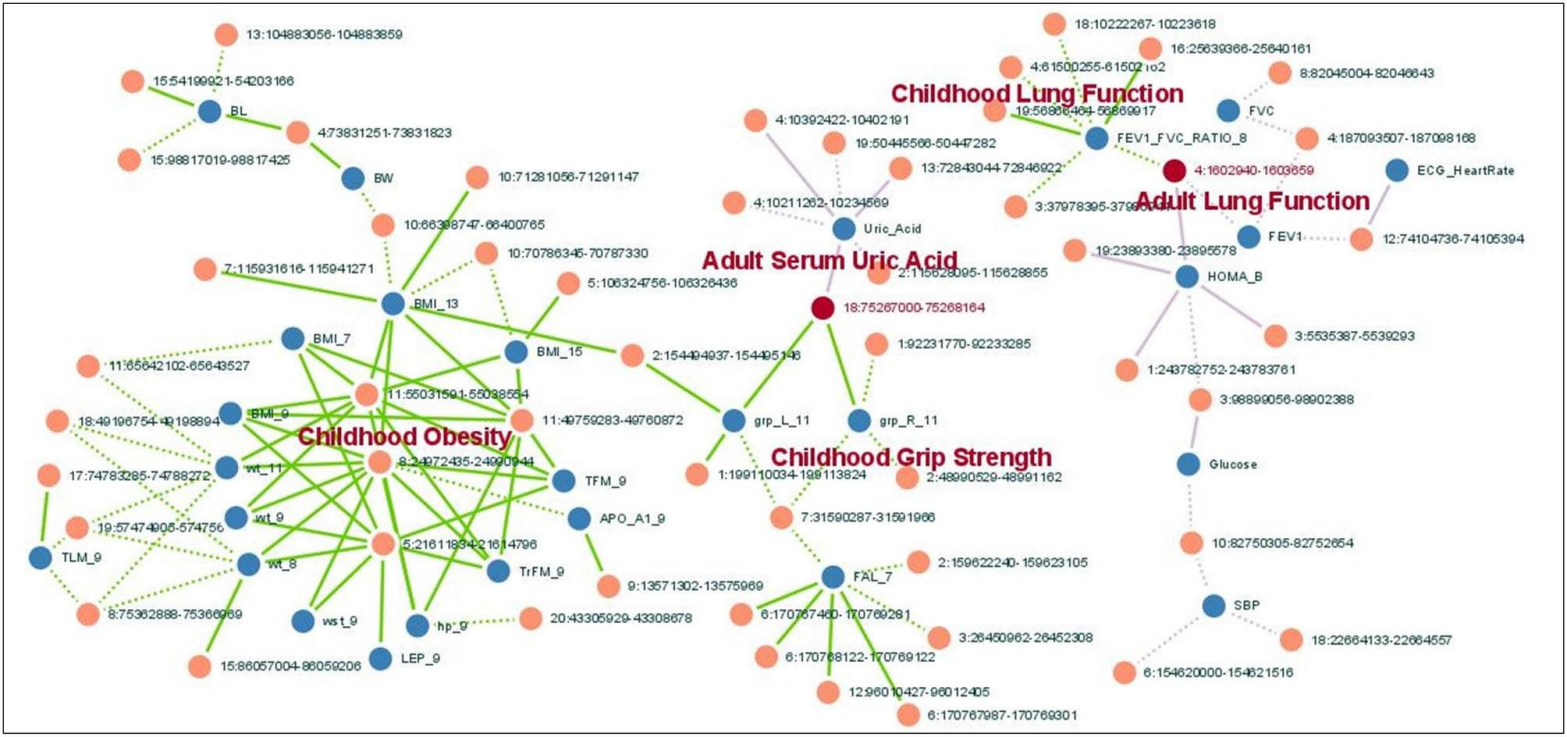
The comprehensive associations between multiple childhood to adult phenotypes based on pleiotropy of CNDs. Phenotypes (blue nodes) and copy number deletions (orange nodes) are connected by edges if they are associated with at least sub-threshold significance (P < 10^− 3^) at TwinsUK(purple edges) and ALSPAC(green edges) cohorts with positive (continuous line) or negative (dotted line) β values. Two CND nodes that connect categories of phenotypes are colored with red

The comprehensive exploration of the associations between multiple phenotypes from childhood to adult and CNDs enabled us to investigate the pleiotropy of TADs by network analysis. The full network (Supplementary Fig. S4) illustrates that adult and childhood phenotypes are generally distinct, suggesting that adult and childhood phenotypes are distinct with respect to TADs. The largest connected core of the full network is shown in Fig. 5. Adult and childhood lung functions are connected by 719 bp deletion on chromosome 4 (1,602,940–1,603,659) associated with obstructive lung functions (FEV1/FVC ratio in childhood and FEV1 in adult). It is concordant with the previous epidemiologic study reporting that being in the lowest quartile for lung function at age 7 might have long-term consequences for the development of COPD by middle age [31]. A 1.2 kb deletion on chromosome 18 (75,267,000–75,268,164) was associated with both elevated adult serum uric acid levels and stronger grip strength in children. Epidemiologic data suggests the association of hand grip strength, a marker of sarcopenia, and serum uric acid levels in middle aged adults [32], and it warrants further study if muscle mass in children predicts serum uric acid levels in adults.

## Discussion

We reanalyzed UK10K WGS cohorts’ data to uncover copy number deletions associated with 60 quantitative traits and found 23 genome-wide significant associations at 13 copy number deletions. Some of the associations replicate previously suggested links between copy number deletions and quantitative traits: the uric acid association signals around the *SLC2A9* locus or childhood obesity related 11q11 region. Others such as red blood cell trait associations are found in regions tagged by red cell traits associated SNVs from cohorts with huge number of subjects with microarray data. The others are novel associations of copy number deletions with traits such as sodium, HDL cholesterol, and lean body mass that require further validation in separate cohorts. We also observed pleiotropy of common copy number deletions that might underlie complex epidemiological associations.

One of the strengths of the study is that we annotated copy number deletions with dbVar reference IDs and population allele frequencies reported in gnomAD structural variation (SV) database with definite breakpoint positions. Since the first descriptions of the genome-wide presence of copy number variations(CNV) in the human genome in year 2004 [1, 2], attempts have been made to build public CNV databases [3]. However, compared to SNPs or Indels that are well curated in dbSNP database with non-redundant and unique Reference SNP (rs) numbers, the mutational spectra of SVs are only recently beginning to be reported based on high-depth whole genome sequencing [9, 10]. As WGS is rapidly becoming the predominant technology in large-scale human studies with progressively decreasing sequencing cost, we expect that trait or disease mapping studies involving structural variations will report reference IDs such as dbVar IDs, enabling curated databases of phenotype related structural variations.

Another strength of the study is that we analyzed association signals by combining SNVs and copy number deletions. The combined analysis found long-ranging haplotype blocks that overlap with regulatory region or gene clusters. For example, around the *SLC2A9* gene on chromosome 4 with very high association signal with serum uric acid [33], nssv15888957 formed a 254 kb-ranging haplotype block in strong linkage disequilibrium with 224 SNVs (R2 > 0.8) spanning putative enhancer region. With respect to childhood obesity, we found several haplotype blocks of CNDs and SNVs that span multiple olfactory receptor gene clusters. Considering studies that elevated BMI is known to be associated with olfactory dysfunction and mouse experiment suggesting that ectopic olfactory receptor activation reverses obesity, the existence of early obesity related CNDs and SNVs in strong LD in 11q11 olfactory gene cluster region might corroborate the role of olfactory dysfunction in obesity [34, 35].

We also found novel trait-associated CNDs in functionally relevant genes which had no previous reports of associations with SNVs or Indels. For example, CND in Intron 1 of the *SGK1* gene (nssv15913056) was associated with elevated sodium levels. *SGK1* is a gene that plays an important role in regulating sodium concentration, and it is involved in the regulation of sodium reabsorption by modulating the activity of the epithelial sodium channel (ENaC) in the distal tubules of the kidney [36]. However, no sodium level associated SNVs or Indels at or near *SGK1* gene have been registered in the GWAS catalogue (https://www.ebi.ac.uk/gwas/) database despite the definite involvement of *SGK1* in sodium metabolism. Further human genetic epidemiologic studies that validate the novel associations in the present study are warranted.

The majority (97.5%) out of 161 Trait-Associated copy number Deletions (TADs) was placed in non-coding regions such as introns or intergenic areas. This genomic impact of common copy number deletions, or the bias away from genes, is consistent with study by Conrad et al. that reported 95.3% of copy number deletions were placed in non-coding regions [37]. Out of 60 TADs annotated in gene regions, 56 TADs were in introns and structural variation in intron might be associated with gene expression (CNV-eQTL) [38] or regulate genes with tissue-specific activity [39]. It is interesting that 42% of TADs were found in non-enhancer intergenic regions and what further genomic features are associated with quantitative traits is the subject of further study.

The study is unique in that it analyzed both adult women and children’s cohorts and we could study the pleiotropy of CNDs with respect to adult and childhood traits. For example, nssv15897230 was associated with childhood sarcopenia and poor lung function in adults that is found in previous epidemiologic studies [30]. In the pleiotropy analysis, 719 bp deletion on chromosome 4 (dbVar ID: nssv15887243, 4:1602940–1,603,659) was associated with obstructive lung functions in childhood and adults (FEV1/FVC ratio in childhood and FEV1 in adult). The longitudinal associations between childhood traits and their impacts on disease in later adult life are important epidemiological questions and further mendelian randomization studies using SNVs and copy number deletions profiled in UK10K WGS cohorts may give further insight.

There are several limitations of the study: (1) The study included relatively moderate number of subjects, (2) only the associations with traits were analyzed because more clinically relevant phenotypes like disease were not available, (3) the replication study was limited as the two cohorts, TwinsUK and ALSPAC, represented two clinically distinct subject groups, middle-aged women versus children, and (4) genome-wide or experiment-wide significant associations need further replicate validation analyses and the pleiotropy analysis could contain false positive findings that require attention in interpretation. With the prospect that millions of genomes will be sequenced in the coming years from national initiatives we expect there will be available replicate WGS cohorts with high-confidence SV calls.

In conclusion, the reanalysis of UK10K WGS cohorts with combined analysis of SNVs and copy number deletions uncovered multiple novel and replicating associations with multiple quantitative traits. The recent explosive growth in whole genome sequence data from multiple biobanks that call both SNVs and structural variations might enable a comprehensive understanding into the genotype to phenotype relationships.

### Electronic supplementary material

Below is the link to the electronic supplementary material.

Supplementary Material 1

Supplementary Material 2

Supplementary Material 3

Supplementary Material 4

Supplementary Material 5

Supplementary Material 6

Supplementary Material 7

Supplementary Material 8

Supplementary Material 9

Supplementary Material 10

## Data Availability

The datasets supporting the conclusions of this article are included within the article and the supplementary materials. Any additional data are available on request to the corresponding author.
