# Supplementary material for: Exploring quantitative traits-associated copy number deletions through reanalysis of UK10K consortium whole genome sequencing cohorts
Source: BMC Genomics. 2023 Dec 18;24:787. doi: 10.1186/s12864-023-09903-3 (PMC10729411; doi:10.1186/s12864-023-09903-3)
Supplement: Supplementary file 1 — Supplementary Material 1 [file 12864_2023_9903_MOESM1_ESM.docx]

**Supplementary materials.**

**Supplementary Table S1.** The list of 48 and 42 phenotypes in TwinsUK and ALSPAC cohort, respectively, and 30 shared phenotypes.

**Supplementary Table S2.** The full list of 18,739 copy number deletions called by GenomeSTRiP with allele frequency information at TwinsUK and ALSPAC cohorts.

**Supplementary Table S3.** The full list of 1,222 and 1,211 common copy number deletions for association analysis in the TwinsUK and ALSPAC cohorts after filtering out variants with a minor allele frequency < 0.05 or Hardy-Weinberg Equilibrium P<1.0×10^-6^.

**Supplementary Table S4.** The GWAS summary statistics for phenotypes in TwinsUK and ALSPAC cohort.

**Supplementary Table S5.** The list of 239 associations of trait-associated 161 copy number deletions (TADs) associated with any phenotypes with at least sub-threshold significance (P < 10^-3^).

**Supplementary Table S6.** The list of 161 Trait-Associated copy number Deletions (TADs) and detailed annotation of deletions in gene regions.

**Supplementary Figure S1.** Manhattan plot of SNVs and Indels associated with uric acid levels in TwinsUK cohort confirming the significant peak on the chromosome 4.

**Supplementary Figure S2.** Manhattan plot of SNVs and Indels with HDL cholesterol levels.

**Supplementary Figure S3.** Fine mapping of SNVs around nssv15893432 associated with total lean mass. The start position of nssv15893432 is marked with diamond.

**Supplementary Figure S4.** Fine mapping of SNVs around nssv15922542 associated with MCH. The start position of nssv15922542 is marked with diamond.

**Supplementary Figure S5.** Fine mapping of SNVs around nssv15813226 associated with MCV. The start position of nssv15813226 is marked with diamond.

**Supplementary Figure S6.** Fine mapping of SNVs around nssv15913056 associated with serum sodium levels. The start position of nssv15913056 is marked with diamond.

**Supplementary Figure S7.** Fine mapping of SNVs around nssv15849193 associated with childhood LDL. The start position of nssv15849193 is marked with diamond.

**Supplementary Figure S8.** The pleiotropy of nssv15897230 which was associated with reduced lung function (FVC and FEV1) in adult women and children was also associated with lower total lean mass and body mass indices in children, suggesting the genetic link between childhood sarcopenia and adult poor lung function.

**Supplementary Figure S9.** The comprehensive map of the associations between multiple childhood to adult phenotypes based on pleiotropy of CNDs.
