# Supplementary material for: Exploring quantitative traits-associated copy number deletions through reanalysis of UK10K consortium whole genome sequencing cohorts
Source: BMC Genomics. 2023 Dec 18;24:787. doi: 10.1186/s12864-023-09903-3 (PMC10729411; doi:10.1186/s12864-023-09903-3)
Supplement: Supplementary file 2 — Supplementary Material 2 [file 12864_2023_9903_MOESM2_ESM.pptx]

## Slide 1
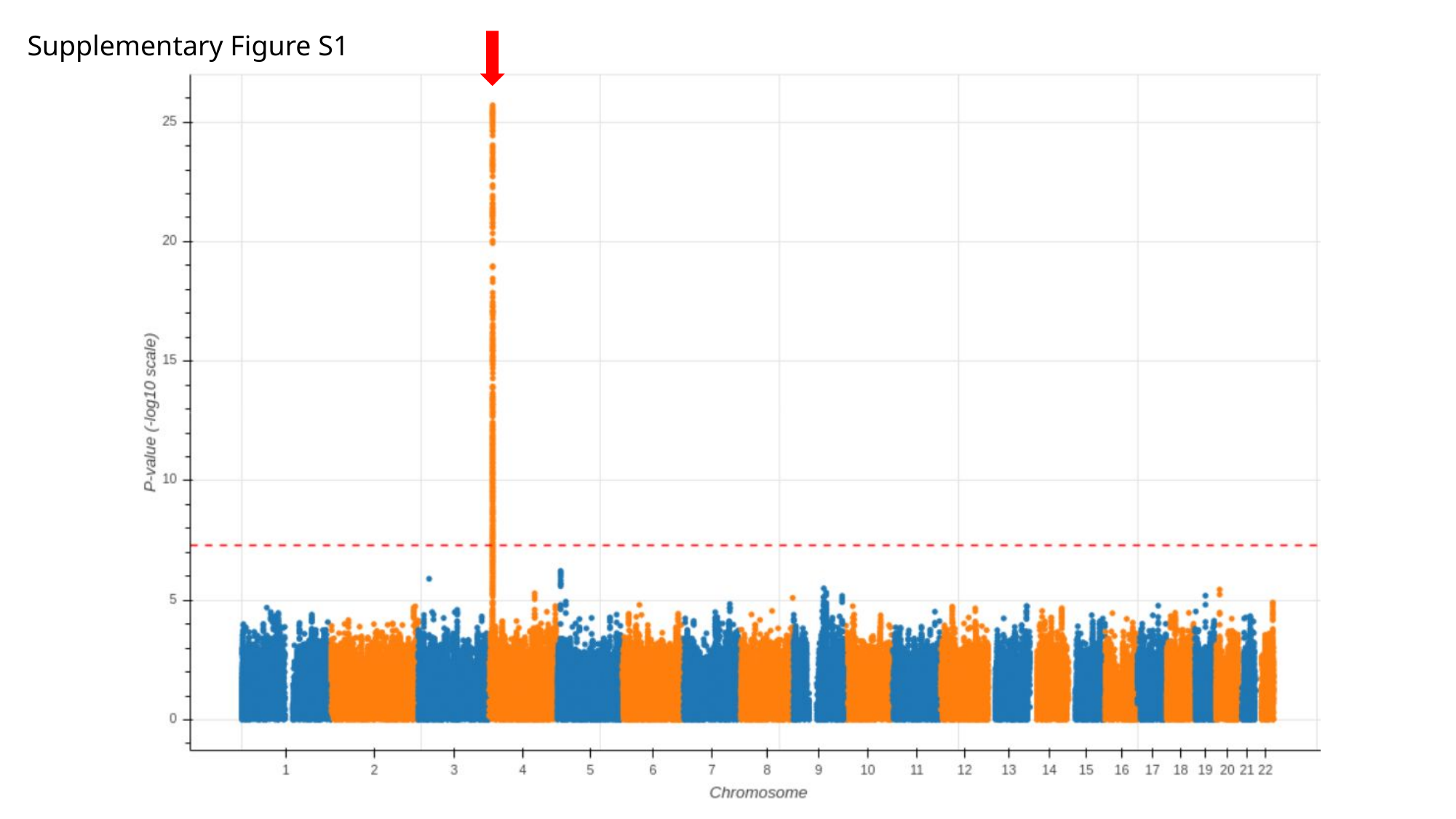

Supplementary Figure S1

## Slide 2
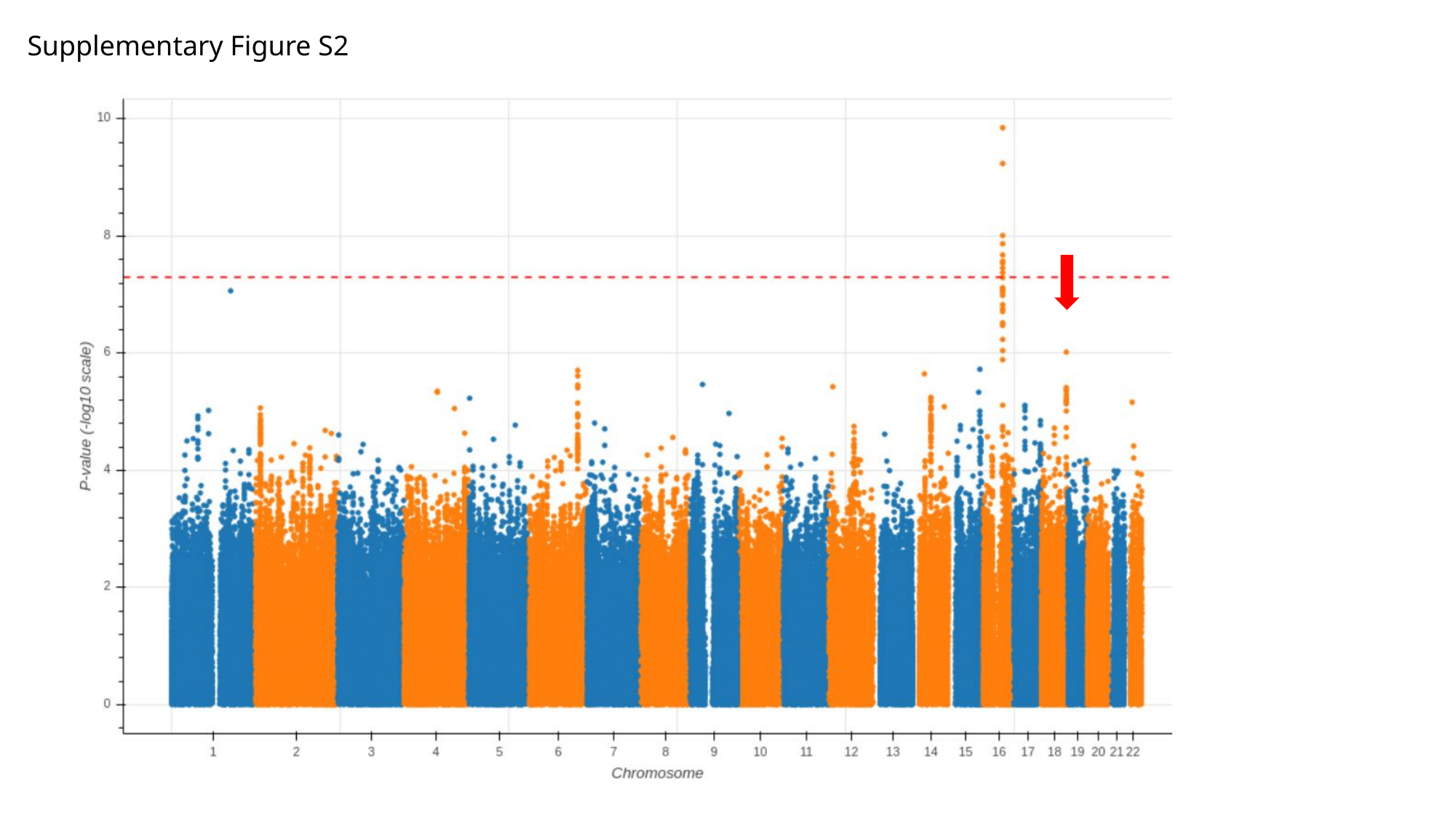

Supplementary Figure S2

## Slide 3
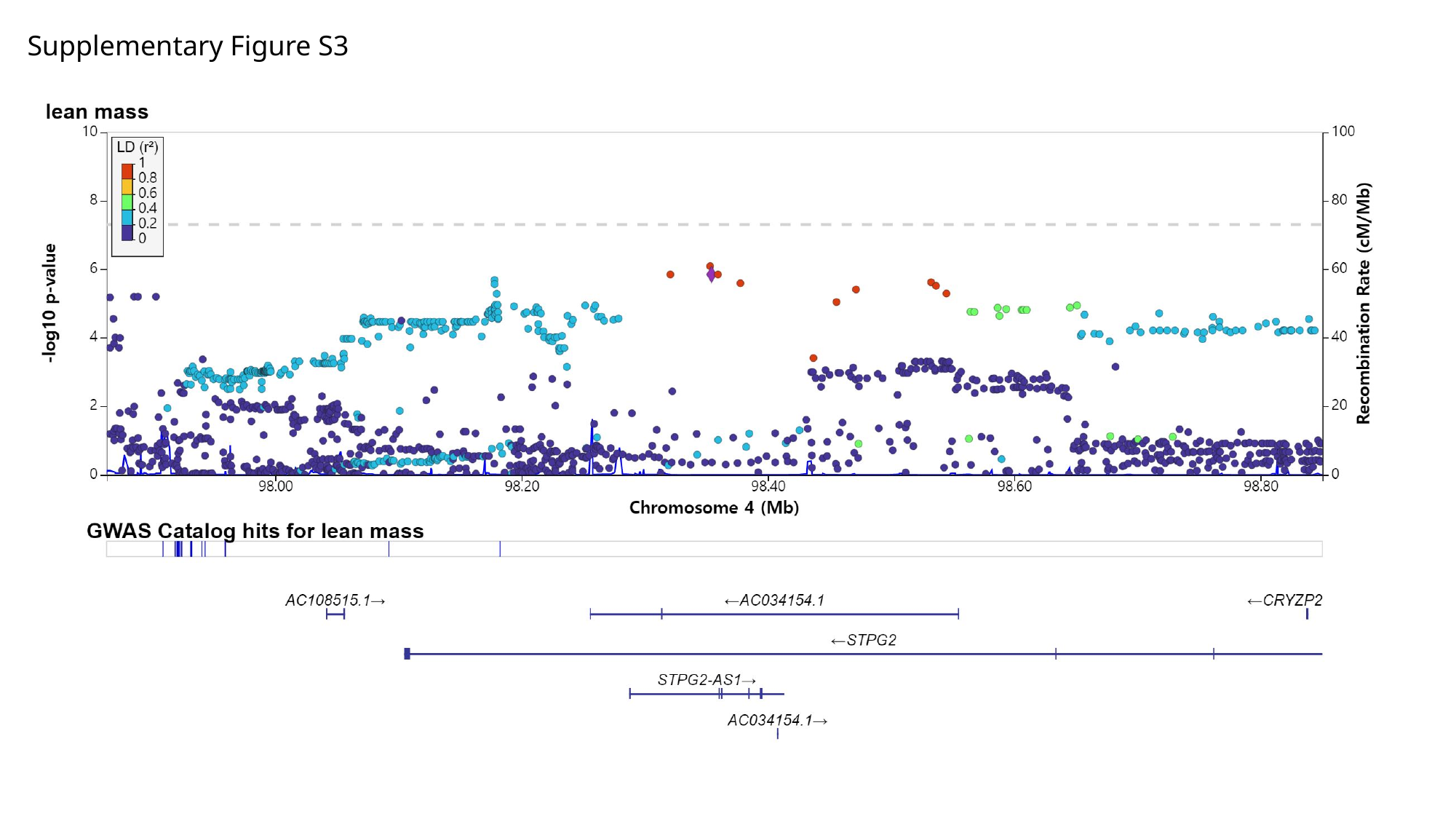

Supplementary Figure S3

## Slide 4
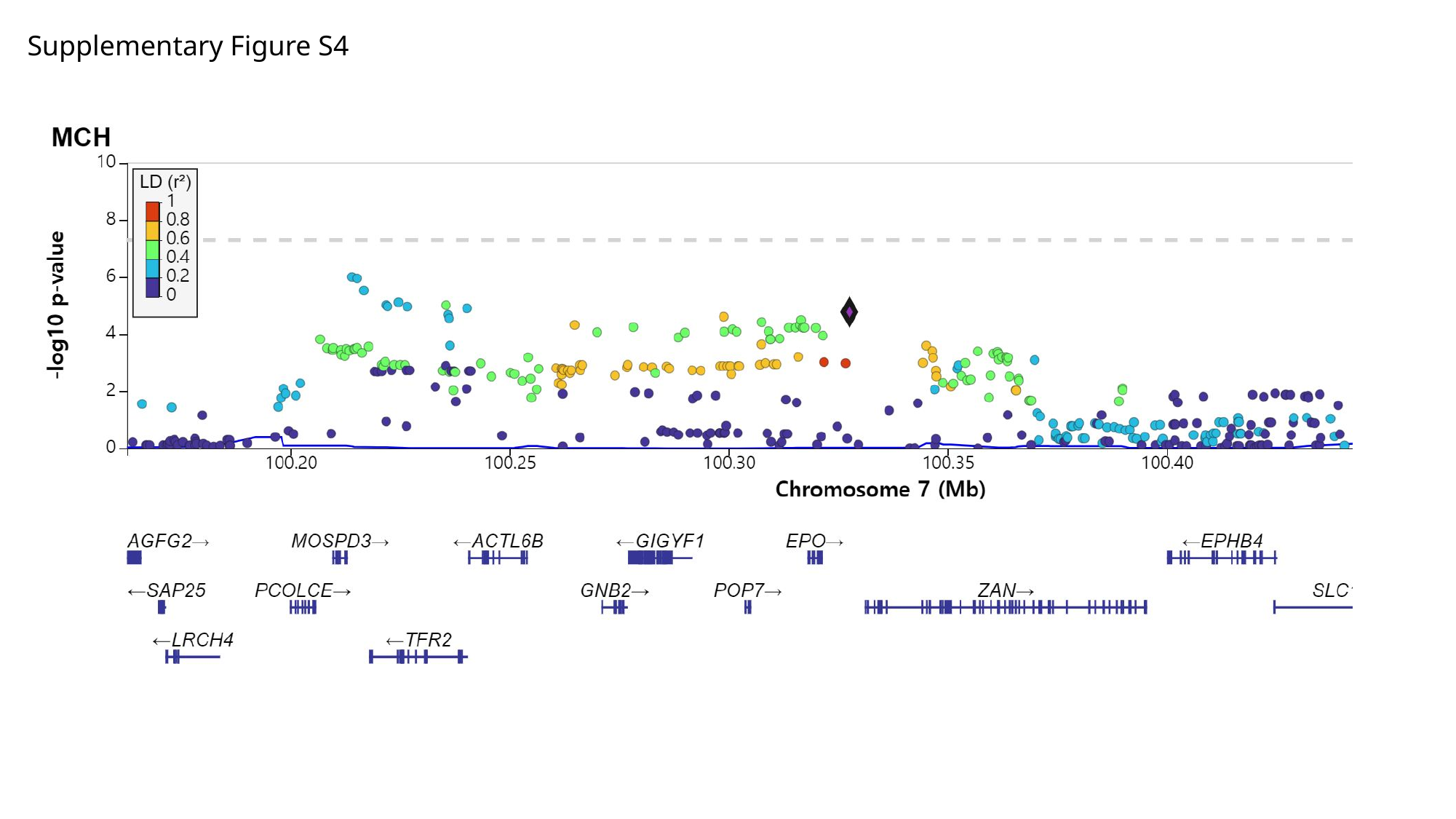

Supplementary Figure S4

## Slide 5
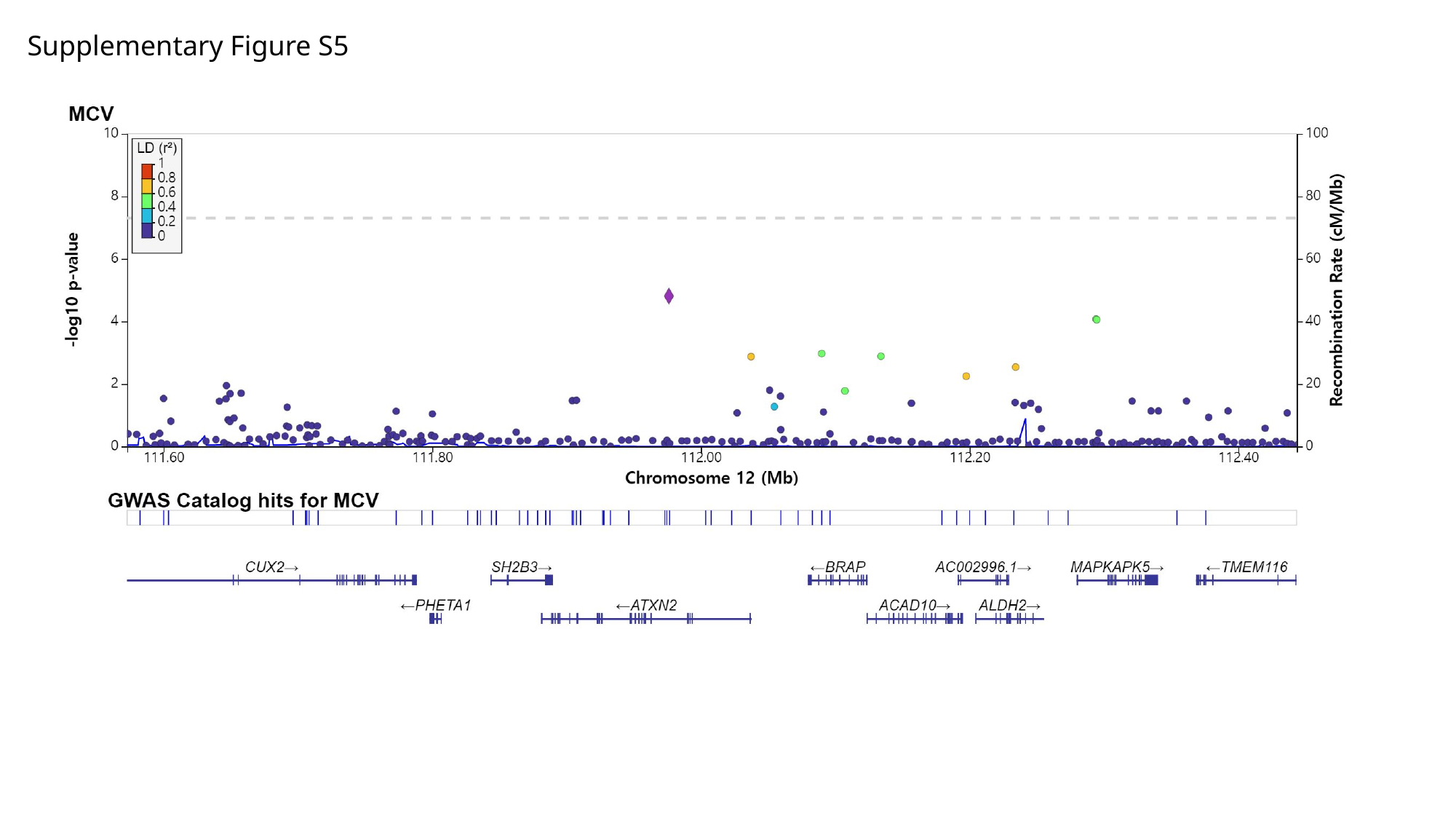

Supplementary Figure S5

## Slide 6
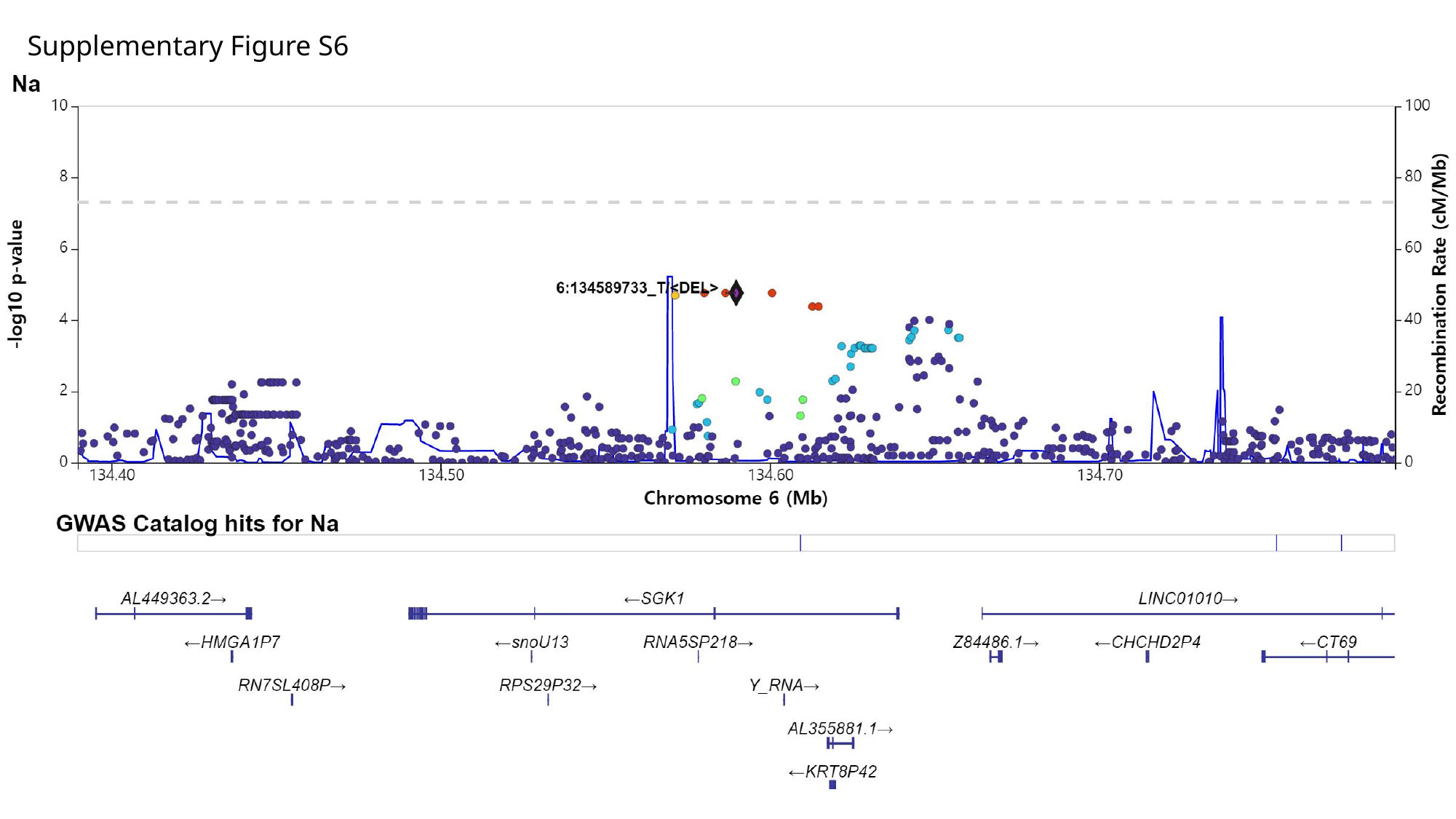

Supplementary Figure S6

## Slide 7
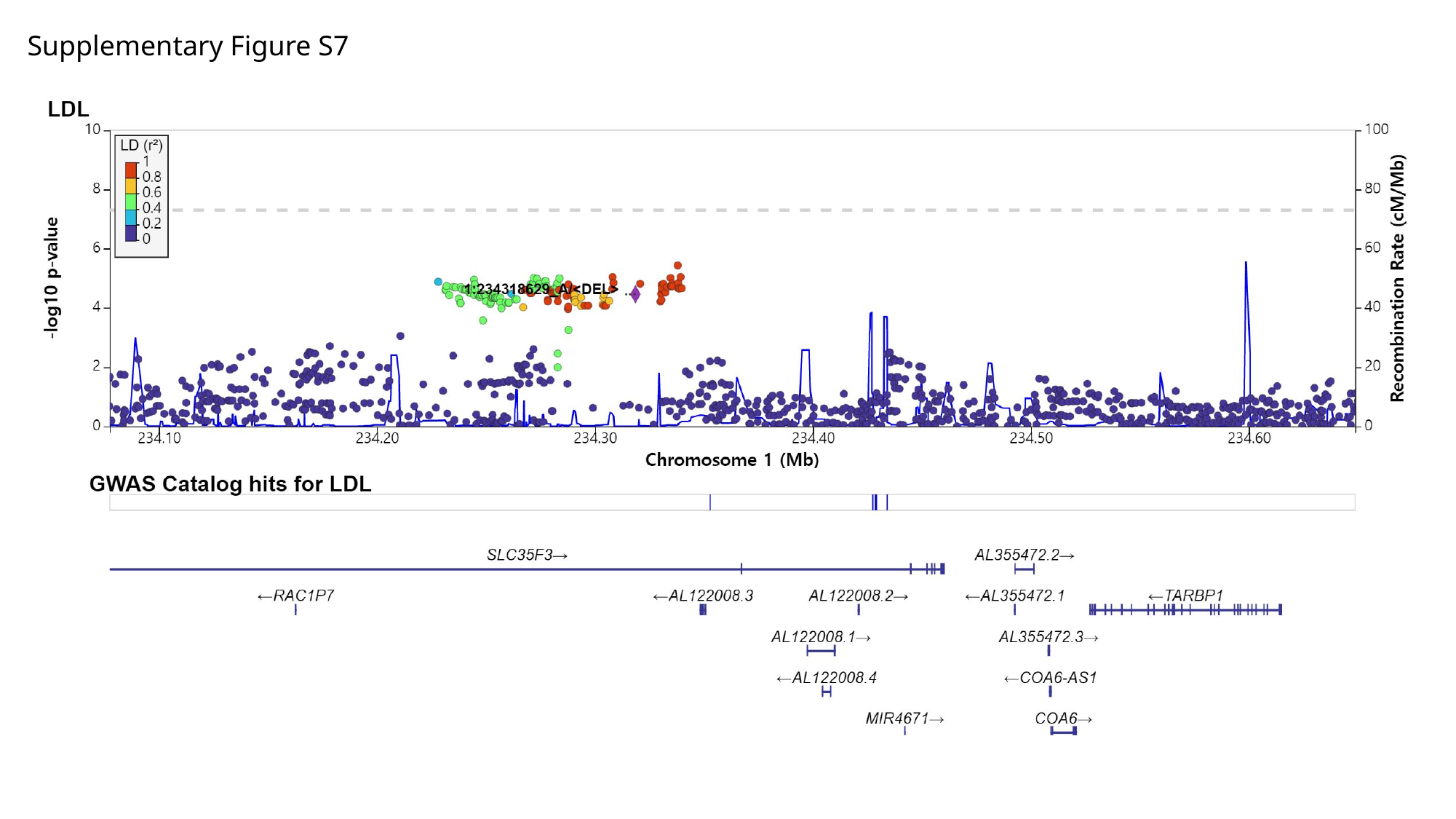

Supplementary Figure S7
