# Supplementary material for: Exploring quantitative traits-associated copy number deletions through reanalysis of UK10K consortium whole genome sequencing cohorts
Source: BMC Genomics. 2023 Dec 18;24:787. doi: 10.1186/s12864-023-09903-3 (PMC10729411; doi:10.1186/s12864-023-09903-3)
Supplement: Supplementary file 3 — Supplementary Material 3 [file 12864_2023_9903_MOESM3_ESM.pptx]

## Slide 1
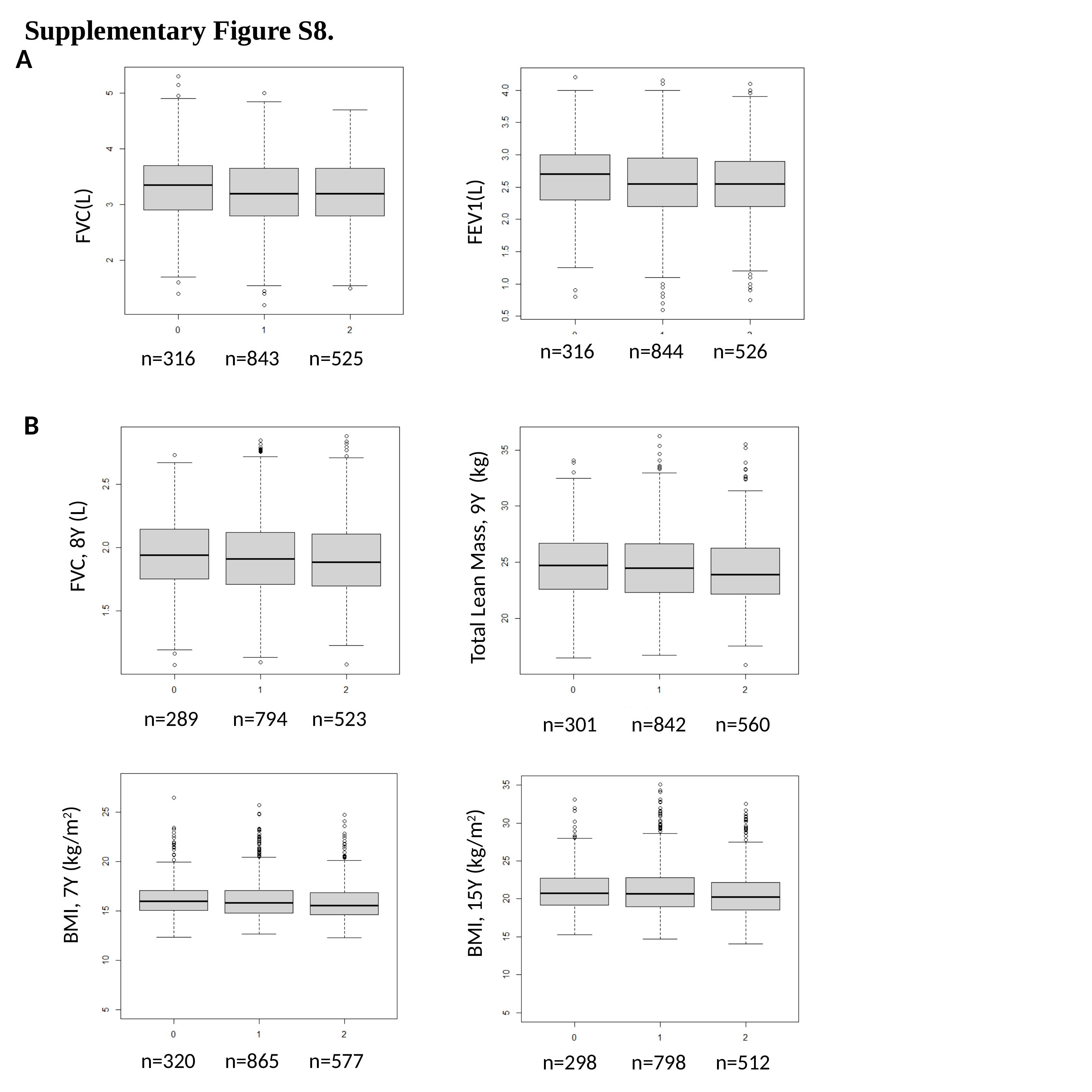

Supplementary Figure S8.
A
FVC(L)
FEV1(L)
n=316 n=844 n=526
n=316 n=843 n=525
FVC, 8Y (L)
B
Total Lean Mass, 9Y (kg)
n=289 n=794 n=523
n=301 n=842 n=560
BMI, 7Y (kg/m2)
BMI, 15Y (kg/m2)
n=320 n=865 n=577
n=298 n=798 n=512
