# Supplementary figures and images for: Exploring quantitative traits-associated copy number deletions through reanalysis of UK10K consortium whole genome sequencing cohorts
Source: BMC Genomics. 2023 Dec 18;24:787. doi: 10.1186/s12864-023-09903-3 (PMC10729411; doi:10.1186/s12864-023-09903-3)

Supplementary Figure S9

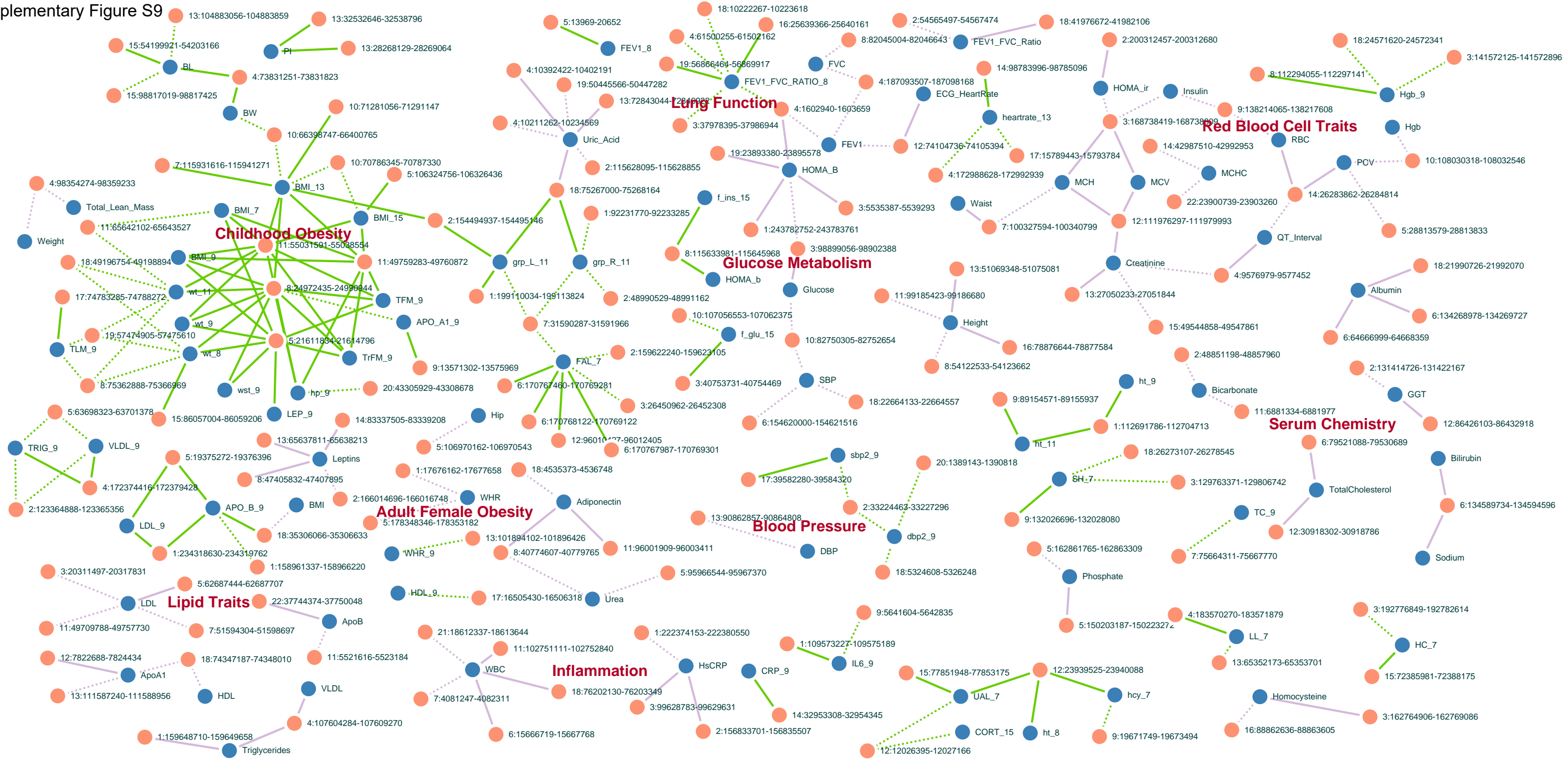

Supplement: Supplementary file 4 — Supplementary Material 4 [file 12864_2023_9903_MOESM4_ESM.pdf]
